# Analysis of the Effect of Human Type I Collagen-Derived Peptide on Bone Regenerative Capacity and Comparison with Various Collagen Materials In Vivo

**DOI:** 10.3390/medicina61010057

**Published:** 2025-01-02

**Authors:** Tatsunori Asakura, Tran Thi Thuy Diep, Yuta Ueda, Aoi Yamada, Takahiro Tsuzuno, Naoki Takahashi, Masayuki Miyata, Koichi Tabeta, Masaki Nagata, Ken Matsuda

**Affiliations:** 1 Department of Plastic and Reconstructive Surgery, Niigata University Graduate School of Medical and Dental Sciences, Niigata 951-8510, Japan; 2 Division of Periodontology, Department of Oral Biological Science, Niigata University Graduate School of Medical and Dental Sciences, Niigata 951-8514, Japan; 3 Division of Pioneering Advanced Therapeutics, Niigata University Medical and Dental Hospital, Niigata 951-8520, Japan

**Keywords:** type I collagen, bone regeneration, RGD motifs, recombinant peptides, scaffolding material, mesenchymal stem cell (MSC)

## Abstract

*Background and Objectives*: Autologous bone grafting is the first choice for reconstructive surgery in bone defects due to trauma or malignant tumors. However, there is an increasing demand for minimally invasive alternatives involving bone regeneration using artificial materials. Biomimetic materials that replicate the body’s microscopic structure, such as Cellnest^®^, are gaining attention. Cellnest is a xeno-free recombinant peptide based on human type I collagen, containing a rich Arg-Gly-Asp (RGD) motif related to cell adhesion. The aim of this study was to compare the effects of Cellnest with existing collagen materials (Pelnac^®^, Integra^®^, Terudermis^®^) on bone regeneration and elucidate the underlying mechanisms. *Materials and Methods*: In vivo experiments involved a rat model of calvarial bone defects, in which Cellnest and other collagen materials were implanted into the defect area. Bone formation was assessed after 4 weeks using micro-computed tomography (micro-CT) and histological analysis. In vitro experiments included the 3-(4,5-dimethylthiazol-2-yl)-2,5-diphenyltetrazolium bromide (MTT), adhesion, and migration assays, and a real-time polymerase chain reaction using rapidly expanding cells (RECs) to explore the mechanisms of Cellnest’s bone regenerative capacity. *Results*: The micro-CT analysis showed that the regenerated bone area was significantly greater in the Cellnest group (72.3%) than in the Pelnac^®^ (25.5%), Integra^®^ (31.6%), and Terudermis^®^ (38.3%) groups. The histological analysis confirmed similar trends, with Cellnest showing 42.2% bone regeneration, outperforming the other materials. The in vitro assays revealed that Cellnest promoted cell proliferation, adhesion, and migration. Gene expression analysis demonstrated that Cellnest significantly increased the levels of the bone formation markers ALP and COL1. *Conclusions*: Cellnest, a human type I collagen-like peptide rich in RGD motifs, enhances bone regeneration by promoting MSC adhesion and migration, and bone formation-related gene expression. The findings suggest its potential as an effective material for bone defect reconstruction.

## 1. Introduction

Bone defects due to trauma or tumors are commonly treated through reconstructive surgery, which generally involves either autologous bone grafting or artificial material use. Autologous bone grafting remains the gold standard, primarily because the graft is derived from the patient’s own tissue, minimizing the risk of infection and presenting a high capacity for bone regeneration. However, this approach has several drawbacks, including the invasiveness of the procedure, complications such as pain and deformation at the donor site, and a limited supply of harvestable bone [1,2,3,4]. The use of artificial bone substitutes offers a potential solution, especially for elderly patients and children, as it eliminates the need for invasive donor site surgery. Despite these advantages, there is no universally accepted artificial bone grafting method, highlighting the need for new bone-forming materials that can serve as effective alternatives to autologous grafts, particularly in the context of minimally invasive procedures.

Bone is a complex tissue comprising both organic and inorganic components that not only provide structural support but also help regulate mineral homeostasis. The organic component is predominantly composed of type I collagen, accounting for about 90%, with the remainder made up of non-collagenous proteins, lipids, and water [5]. Given the prominence of type I collagen as the primary organic component, collagen-based substitutes have gained substantial interest in bone tissue engineering [6,7,8]. Natural type I collagen has been reported to enhance cell adhesion and promote osteogenic differentiation, making it a valuable material for bone regeneration [9,10,11].

Cellnest^®^, a novel recombinant peptide (RCP) designed to mimic type I collagen, contains 12 Arg-Gly-Asp (RGD) motifs, which exhibit a strong affinity for integrins, receptors involved in promoting cell adhesion and growth [12]. This RGD-rich structure endows Cellnest with superior cell adhesion properties. Furthermore, Cellnest is a xeno-free product, containing no animal-derived components, which minimizes the risk of disease transmission and makes it a safer option [13]. Its versatility also allows for easy processing into various forms, such as sheets, granules, and films. An ideal scaffold for bone regeneration should be bioabsorbable, allowing for gradual replacement by new bone tissue; Cellnest meets this criterion, being susceptible to enzymatic degradation [14,15,16,17,18,19]. In contrast, existing collagen materials are derived from animal sources, which can carry foreign antigens and exhibit some level of immunogenicity [20]. Recently, artificial bone materials incorporating growth factors and extracellular matrices have been developed, and their ability to promote bone regeneration has been enhanced [21,22]. However, there are concerns about their long-term stability and integration with bone remains [23]. Cellnest has the potential to yield mature regenerated bone owing to its high osteoconductivity. Currently, no studies have compared the bone-forming ability of Cellnest with that of existing collagen-based materials, creating a gap in our understanding of their relative efficacy. Additionally, while some studies have explored the use of recombinant collagen peptides (RCPs) in combination with mesenchymal stem cells (MSCs) [12], the presence of other cell types in MSC preparations can introduce variability in their regenerative properties, complicating the assessment of efficacy.

In this study, we aimed to fill this gap by comparing the bone formation capacity of Cellnest, formulated as a sheet, with that of commercially available collagen sheet materials in a rat calvarial defect model. Furthermore, we investigated the mechanisms underlying bone regeneration through in vitro experiments using highly purified rapidly expanding cells (RECs) [24]. Our results suggest that RGD-rich collagen materials such as Cellnest can regenerate bone more effectively than existing collagen materials, supporting their potential clinical application.

## 2. Materials and Methods

### 2.1. Materials

Cellnest is a biomaterial developed by Fujifilm (Tokyo, Japan). It is synthesized using a plasmid that encodes the RGD motif, followed by the addition of distilled water to create an aqueous solution. This solution is then frozen, dried, and cross-linked in a vacuum oven to form a Cellnest sheet [12]. There are 2 RGD motifs in a single molecule of human type I collagen, whereas there are 12 RGD motifs in a single molecule of Cellnest. Therefore, Cellnest has six-fold more RGD sequences than human type I collagen.

Pelnac^®^ (Gunze Medical, Osaka, Japan), Integra^®^ (Century Medical, Tokyo, Japan), and Terudermis^®^ (ALCARE, Tokyo, Japan) are synthetic heterologous collagen materials approved by the Japanese Pharmaceutical and Medical Device Act for clinical use. Pelnac^®^ is a freeze-dried material primarily composed of porcine tendon atelocollagen, chemically cross-linked to inhibit degradation and prevent shrinkage [25]. Integra^®^ is derived from bovine tendon type I collagen and shark chondroitin-6-sulfate glycosaminoglycan, combined with a silicone-based pseudo-epidermis [26]. Terudermis^®^ consists of heat-denatured bovine dermal type I collagen that has undergone cross-linking via dehydration heat treatment [27]. All these materials are low-antigenicity atelocollagens and are used clinically to form dermal-like tissue [28]. These three types of collagen materials are typically used in clinical practice to produce dermal-like tissue. To investigate the effect of excess or low levels of the RGD motif on bone regeneration, we compared materials made from the same type I collagen as Cellnest.

RECs for the experiments were purchased from Fujifilm. These cells were isolated from human bone marrow using flow cytometry and selective markers. Unlike mesenchymal stem cells obtained using conventional adhesion methods, they contain almost no contaminating cells and can be cultured and expanded over long periods [24]. We predicted that using REC would allow us to analyze the function of MSCs more accurately.

### 2.2. Animals

All experiments were conducted on 6-week-old male Wistar rats weighing 160–180 g (The Jackson Laboratory, Yokohama, Japan). We used male rats because female rats may be affected by endocrine factors related to their sexual cycle. We created a list of target samples, performed a random shuffle, and randomly changed the order of the list. Subsequently, we divided the list into treatment and control groups based on the random list created. The rats were housed in the Niigata University Animal Experiment Center under a 12:12 h light/dark cycle at a standardized temperature and humidity. As this study involved exploratory experiments aimed at providing initial insights useful for further investigations, we used the minimum number of animals feasible from the perspective of animal welfare and with limited resources. This study was conducted following the tenets of the Declaration of Helsinki and was approved by the Institutional Animal Care and Use Committee of Niigata University (approval number: SA01074).

### 2.3. Surgical Procedures

General anesthesia was induced in the rats via isoflurane inhalation, followed by an intraperitoneal injection of a mixture of medetomidine (0.15 mg/kg), midazolam (2 mg/kg), and butorphanol (2.5 mg/kg) at a dose of 0.1 mL per 10 g of body weight. A skin incision was made along the top of the head, and the periosteum was peeled back to expose the parietal bone. A 5 mm diameter bone defect was created using a trephine bur. After placing the Cellnest sheet on the bone defect and confirming that the blood had infiltrated and the material had stabilized, the scalp was slowly returned and sutured with 5-0 nylon thread. This procedure was repeated for the transplantation of other collagen materials. Rats that did not receive an implant were used as the control group. Four weeks after surgery, the rats were euthanized using CO_2_ overdose and cervical dislocation. Samples were collected and fixed in 4% paraformaldehyde phosphate buffer.

### 2.4. Micro-CT Examination

Micro-CT imaging was performed using a SkyScan 1174 device (Bruker, Kontich, Belgium) on the day after sampling. The imaging parameters were 50 kV, 800 μA, 1.4° rotation step, 180° rotation, and a scan time of 30 min, averaging two frames per step. Images were reconstructed into 3D models using the built-in ultra-high-speed image reconstruction software NRecon (version 1.6.9.4). The rate of new bone formation was calculated as the ratio of the area of new bone to the area of the bone defect using ImageJ (version 1.54f).

### 2.5. Histological Analysis

The parietal bones fixed in 4% paraformaldehyde phosphate buffer were degreased with ethanol, decalcified using 10% EDTA at 4 °C for 28 days, and embedded in paraffin. Sections were cut transversely at a thickness of 3 µm and stained with hematoxylin and eosin (H&E). Bone regeneration was quantified by measuring the area of newly formed bone relative to the total bone defect area on high-magnification H&E-stained images using ImageJ.

### 2.6. Cell Culture

RECs were cultured in Dulbecco’s modified Eagle medium (DMEM; Invitrogen Corporation, Paisley, Scotland) supplemented with 10% fetal bovine serum (FBS; Nichirei Bioscience Inc., Tokyo, Japan), antibiotics (10,000 U/mL penicillin and 10,000 μg/mL streptomycin), and basic fibroblast growth factor (bFGF) in a humidified incubator at 37 °C with 5% CO_2_.

### 2.7. 3-(4,5-Dimethylthiazol-2-yl)-2,5-diphenyltetrazolium Bromide Assay

Cell viability following Cellnest stimulation was assessed using the 3-(4,5-dimethylthiazol-2-yl)-2,5-diphenyltetrazolium bromide (MTT; Sigma Aldrich Japan, Tokyo, Japan) assay [29]. RECs at passage three were seeded in a 96-well plate (TPP; Techno Plastic Products, Trasadingen, Switzerland) at a density of 1000 cells per well and treated with Cellnest at concentrations of 2.5, 25, and 250 μg/mL for 48 h. After treatment, 200 μL of MTT was added to each of the well plates, and the samples were incubated for 4 h. The supernatant was removed, and 0.2 mL of dimethyl sulfoxide (DMSO) was added to each well to dissolve the formazan crystals. Cell viability was determined by measuring the optical density (OD) at 570 nm using a microplate reader (SpectraMax ABS Plus; Molecular Devices Japan, Tokyo, Japan). The same procedure was repeated after 72 h of incubation.

### 2.8. Adhesion Assay

A cell adhesion assay was performed using CellTracker™ Green CMFDA dye (Thermo Fisher Scientific Inc., Waltham, MA, USA). A working solution was prepared by mixing 50 mg of CellTracker™ Green CMFDA dye, 10 mL of DMSO, and 10 mL of phosphate-buffered saline (PBS). RECs cultured in a 75 cm^2^ flask were washed with PBS and treated with 2 mL of the CellTracker working solution. After incubation at 37 °C for 15 min, the cells were washed with PBS to remove excess reagent, collected, and seeded in two 96-well plates (1000 cells per well), forming four groups (control, Cellnest 2.5 μg/mL, Cellnest 25 μg/mL, and Cellnest 250 μg/mL). Fluorescence microscopy was conducted at 1 and 2 h using a Keyence microscope (BZ-X710; Keyence, Osaka, Japan). Attached RECs were automatically detected and quantified based on fluorescence values using Fiji software (version 1.54f).

### 2.9. Migration Assay

For the migration assay, 24-well plates were seeded with RECs at a density of 1 × 10^5^ cells/mL (100 μL per well, *n* = 6). After 24 h, a scratch was made across the cell layer with a 200 μL pipette tip to create a “wound,” and an initial photograph was taken using a Keyence microscope (0 h). Photographs were also taken at 6 and 12 h after Cellnest stimulation. Wound closure was analyzed by measuring the remaining area of the scratch using Fiji software.

### 2.10. Real-Time Polymerase Chain Reaction

RECs were seeded at 1 × 10^5^ cells/0.1 mL per well in a 24-well plate and cultured in DMEM supplemented with 10% FBS and 1% antibiotics (penicillin/streptomycin) at 37 °C in a 5% CO_2_ atmosphere. After 24 h, the culture medium was replaced, and the cells were stimulated with Cellnest at concentrations of 2.5, 25, and 250 μg/mL. Untreated cells served as the control group, and all cells were cultured for an additional 24 h. Total RNA was extracted using the RNeasy Mini Kit (Qiagen, Venlo, The Netherlands), and its concentration was determined using NanoDrop 1000 (Thermo Fisher Scientific Inc., Waltham, MA, USA). cDNA was synthesized from RNA using the PrimeScript RT Reagent Kit (Takara Bio Inc., Shiga, Japan). Real-time polymerase chain reaction (PCR) was carried out using the QuantStudio Real-Time PCR System (Thermo Fisher Scientific Inc., Waltham, MA, USA) with PowerUp SYBR Green Master Mix (Thermo Fisher Scientific Inc., Waltham, MA, USA). Data for each sample were normalized to GAPDH expression using the ΔΔ^Ct^ method [30].

### 2.11. Statistical Analysis

All data are presented as mean ± standard error of the mean (SEM). The Kruskal–Wallis test followed by Dunn’s post hoc test was used to compare the effects of different Cellnest concentrations with the control. All statistical analyses were performed using GraphPad Prism 8 (GraphPad Software, Inc., La Jolla, CA, USA), and results with *p* values < 0.05 were considered statistically significant.

## 3. Results

### 3.1. Transplantation of Cellnest and Collagen Materials into a Rat Calvarial Bone Defect Model

**Figure 1:**
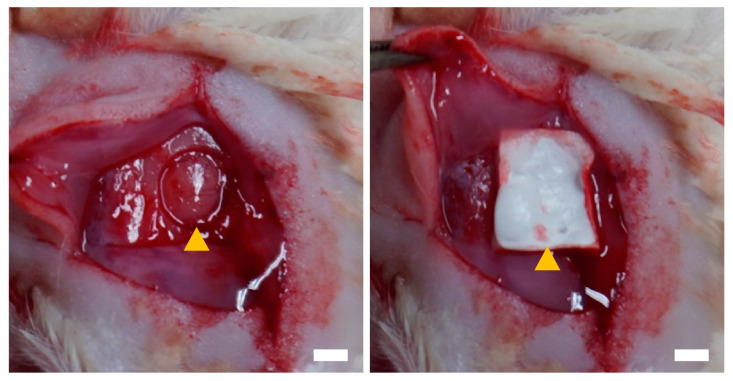
Photographs illustrating the implantation procedure of a Cellnest sheet in a rat calvarial bone defect model. The left panel shows the bone defect before implantation (indicated with the arrowhead) and the right panel shows the site after implantation (indicated with the arrowhead). Scale bar = 3 mm.

**Figure 2:**
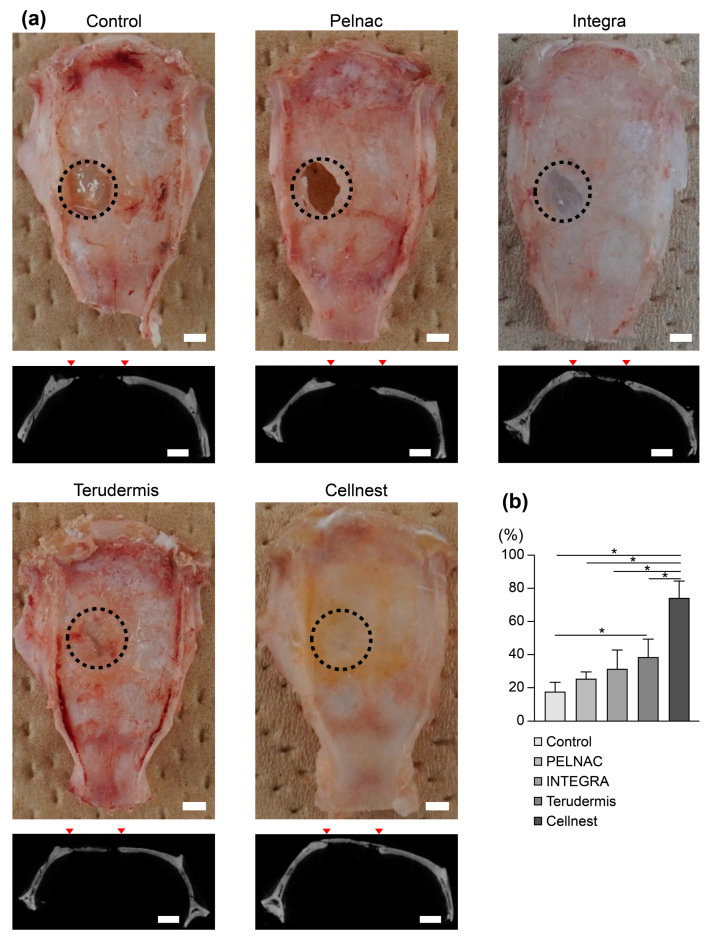
Comparison of bone formation between Cellnest and other collagen materials using micro-CT analysis. (**a**) Upper row displays representative photograph of rat calvaria (dotted lines indicate bone defects). Lower row displays representative micro-CT images of horizontal sections. Between ▼: the border of bone defect. (**b**) Quantification of the bone-forming area. Kruskal–Wallis test and Dunn’s post hoc test (*n* = 3, mean ± SEM) * *p* < 0.05. Scale bar = 2 mm.

To evaluate the effects of Cellnest and other collagen-based materials on bone regeneration, Cellnest and three types of sheet-shaped collagen materials were transplanted into a calvarial bone defect in rats, with a non-transplanted group serving as the control (Figure 1). Gross examination of the control group revealed almost no bone formation, with the defect primarily covered by fibrous connective tissue. In the Cellnest transplant group, bone formation was observed throughout the bone defect area, whereas in the collagen material transplant groups, bone formation was limited to fragments along the defect boundary (Figure 2a).

Micro-CT imaging demonstrated that the Cellnest group exhibited thick, extensive new bone formation, whereas the collagen material groups showed only thin new bone (Figure 2a). Quantitative analysis of the micro-CT images revealed significantly higher bone formation in the Cellnest group than in the control (Figure 2b). Among the collagen groups, only the Terudermis^®^ group showed significantly higher bone formation relative to the control, whereas the Pelnac^®^ and Integra^®^ groups did not show significant differences (Figure 2b). Notably, bone formation in the Cellnest group was significantly greater than that in all other collagen material groups (Figure 2b).

**Figure 3:**
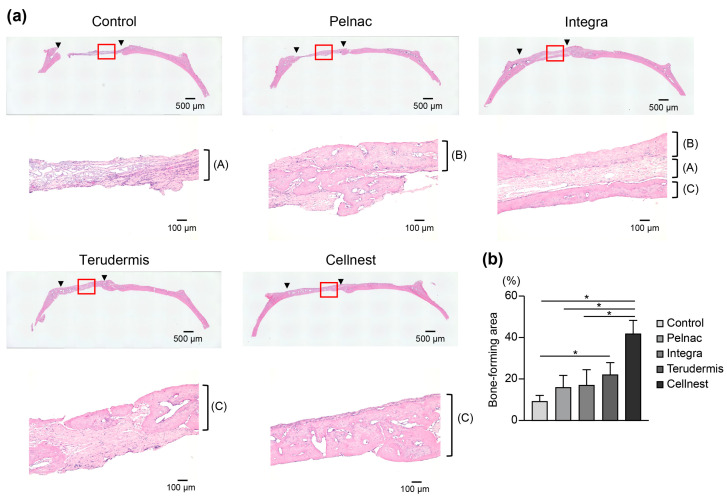
(**a**) Histological images of hematoxylin–eosin-stained specimens of representative samples. Upper row: low magnification, lower row: high magnification in the red square areas. Between ▼: the bone defect, red squares: enlarged area, (A) fibrous tissue, (B) woven bone tissue, (C) new bone tissue. (**b**) Quantification of bone-forming area. Kruskal–Wallis test and Dunn’s post hoc test (*n* = 3, mean ± SEM) * *p* < 0.05.

The histological analysis using H&E staining confirmed that there was almost no new bone formation in the control group, with the defect predominantly filled by fibrous connective tissue. In the collagen material groups, the presence of newly formed tissue varied: some materials showed high fibrous tissue content, while others showed woven bone-like structures. The Cellnest group exhibited some fibrous tissue but predominantly demonstrated mature new bone formation (Figure 3a). Furthermore, while a substantial portion of the other collagen materials remained unabsorbed, most of the Cellnest material was absorbed. Quantitative analysis of bone formation in histological sections showed that the Cellnest group had significantly greater bone formation than the collagen material groups, except the Terudermis^®^ group (Figure 3b). These in vivo results suggest that Cellnest, enriched with RGD motifs, enhances bone formation more effectively than other collagen materials.

### 3.2. Assessment of Cytotoxicity of Cellnest

**Figure 4:**
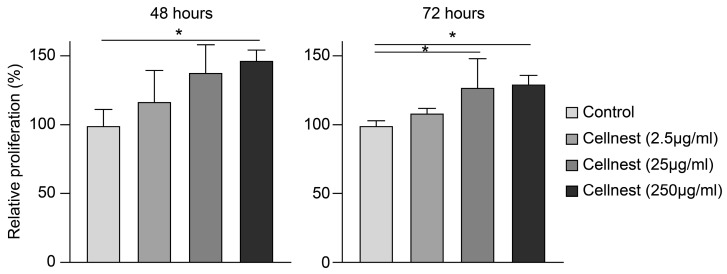
Relative proliferation rate of RECs measured using the MTT assay after Cellnest stimulation for 48 and 72 h (control set to 100%). Data are presented as mean ± SEM (*n* = 5). Statistical analysis was performed using Kruskal–Wallis test with Dunn’s post hoc test. * *p* < 0.05 indicates statistical significance.

Following the transplantation experiment, in which Cellnest demonstrated a capacity to promote bone formation, we further analyzed its effects on cellular function to investigate the underlying mechanism. Cytotoxicity was evaluated using the MTT assay to determine the viability of RECs following Cellnest stimulation. After 48 h, REC proliferation was significantly enhanced in the 250 μg/mL group, and after 72 h, both the 25 μg/mL and 250 μg/mL groups showed increased proliferation. No decrease in cell count was observed at any concentration, indicating that Cellnest is non-cytotoxic and promotes REC proliferation (Figure 4).

### 3.3. Adhesion Assay Analysis

**Figure 5:**
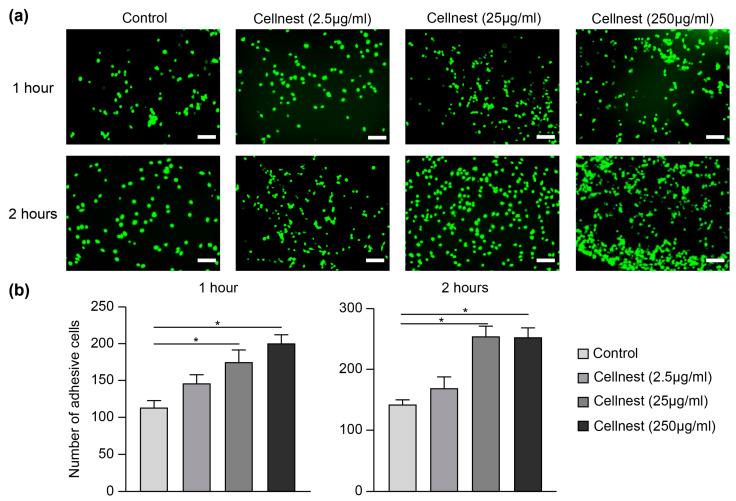
Assessment of REC adhesion. (**a**) Fluorescence images of adherent RECs. Scale bar = 200 μm. (**b**) Quantification of cell adhesion. Data are presented as mean ± SEM (*n* = 5). Statistical analysis was conducted using Kruskal–Wallis test with Dunn’s post hoc test. * *p* < 0.05 indicates statistical significance.

To evaluate Cellnest’s effect on cell adhesion, an important factor for bone regeneration and wound healing, RECs were stimulated with different concentrations of Cellnest. After both 1 and 2 h of stimulation, significant differences in the adhesion rate were observed in the 25 μg/mL and 250 μg/mL groups compared to the control (Figure 5a,b). These results indicate that Cellnest enhances REC adhesion in a time- and concentration-dependent manner.

### 3.4. Migration Assay Analysis

**Figure 6:**
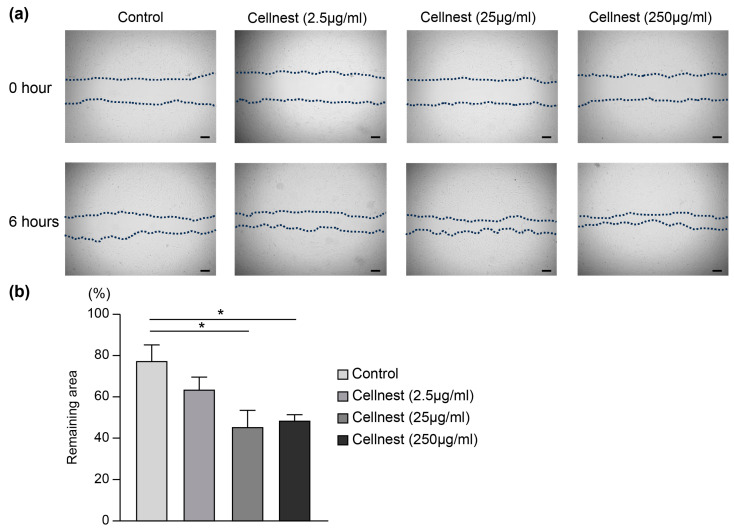
Assessment of REC migration using a scratch assay. (**a**) Representative images of the scratch assay showing the cell-free area bordered by dotted lines. Scale bar = 200 μm. (**b**) Quantification of the remaining cell-free area. Data are presented as mean ± SEM (*n* = 5). * *p* < 0.05 indicates statistical significance.

Given that Cellnest demonstrated enhanced cell adhesion, we conducted a migration assay to evaluate its effect on REC motility. After 6 and 12 h of treatment, both the 25 μg/mL and 250 μg/mL Cellnest groups showed significantly increased migration compared to the control group (Figure 6a,b). These findings suggest that Cellnest not only enhances cell adhesion but also promotes cell migration, which are critical properties for bone regeneration and wound healing.

### 3.5. Real-Time PCR Analysis

**Figure 7:**
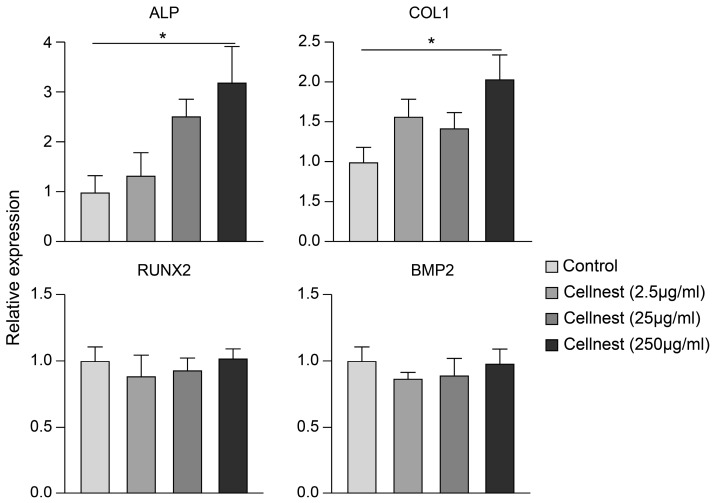
Relative expression levels of osteogenesis-related genes (*ALP*, *COL1*, *RUNX2*, and *BMP2*) following Cellnest stimulation (control was set to 1). Data are presented as mean ± SEM (*n* = 3). Statistical analysis was performed using the Kruskal–Wallis test with Dunn’s post hoc test. * *p* < 0.05 indicates statistical significance.

To further assess the effect of Cellnest on the osteogenic differentiation of mesenchymal stem cells (MSCs), we performed real-time PCR. The expression of alkaline phosphatase (ALP) and collagen type 1 (COL1), both of which are representative osteogenic markers, was significantly upregulated in the 250 μg/mL Cellnest group compared with that in the control group (Figure 7). These results suggest that Cellnest facilitates the differentiation of MSCs into osteoblasts, contributing to enhanced bone regeneration.

## 4. Discussion

Numerous studies have demonstrated the efficacy of calcium phosphate-based materials, such as hydroxyapatite and β-TCP, as bone-forming scaffolds [31,32,33,34,35,36]. Recently, interest has increased in using biomimetic materials in bone tissue engineering [9]. Pountos et al. showed that bioactive peptides promote bone healing reactions [37], whereas Szwed-Georgiou et al. reported that the practical benefit of using these peptides is that they can be produced with precise control of their chemical structure [38]. Collagen, the main organic component of bone tissue, is the most abundant protein in the body; because of its low immunogenicity, it is highly safe and has attracted attention as a scaffold for bone regeneration [39]. However, as pure collagen alone does not have osteoinductive properties, it is not possible to obtain satisfactory bone regeneration outcomes [40,41]. To address these limitations, we focused on Cellnest, a recombinant human type I collagen peptide rich in RGD motifs mainly through interactions with integrin receptors [42], which are known to facilitate cell adhesion [18], and assessed its osteogenic potential. To our knowledge, this is the first study comparing the osteogenic capacity of Cellnest with that of existing commercial collagen-based materials.

In our in vivo experiments, the Cellnest transplant group demonstrated significantly higher new bone formation rates than all collagen material groups, as evidenced using micro-CT imaging. Although we found a significant difference in the bone-forming area, studies are needed to clarify the difference in volume. Similarly, histological analysis of H&E-stained sections showed that bone formation in the Cellnest group was significantly greater than that in the Pelnac^®^ and Integra^®^ groups. These findings suggest that the higher the density of RGD motifs in a material, the more enhanced is its role in promoting bone formation. Bone-forming scaffolds must undergo biodegradation at an appropriate rate to support physiological cell activity, minimize interference with new bone formation, and prevent adverse effects from the release of degradation products [9,43,44,45,46,47,48,49]. In our study, the high density of RGD motifs in Cellnest, combined with its favorable absorbability, likely contributed to its enhanced osteogenic potential.

Based on these promising in vivo results, we sought to investigate the underlying mechanism of bone formation facilitated by Cellnest using in vitro experiments with RECs. The MTT assay showed that Cellnest stimulation promoted REC proliferation. In the adhesion assay, Cellnest at concentrations of 25 and 250 μg/mL significantly increased REC adhesion, consistent with the findings of a previous study reporting the promotion of cell proliferation and adhesion by RGD peptides [50]. Additionally, in the migration assay, as the concentration of Cellnest was increased, the migration of RECs was promoted, suggesting a correlation between the density of the RGD motif and migration ability. The effect of Cellnest on osteogenic differentiation was further assessed using real-time PCR. The expression of *ALP* and *COL1*, which are key osteogenic markers, was significantly upregulated in the 250 μg/mL group. Based on these results and those of in vivo assays, the Cellnest stimulation of MSCs may have induced bone differentiation. These results suggest that an increased density of RGD motifs in human type I collagen improves its effectiveness as an osteogenic scaffold.

However, the expression of bone formation markers such as RUNX2 and BMP2 did not show a significant increase. Previous studies have indicated that RUNX2 expression occurs at a later stage compared to COL1 expression during osteogenesis [51,52]. Therefore, it is possible that the expression of RUNX2 was not detectable within the timeframe of this study. In future studies, it will be necessary to reassess the expression of osteogenesis-related genes over a longer stimulation period.

This study has a few limitations. First, the experimental period for animal studies was relatively short. Some reports suggest that an 8-week period is more suitable for evaluating bone regeneration in implanted materials [53], and our analysis may not have been sufficient to capture changes over time. Moreover, we used the onlay grafting method, which is straightforward and suitable for clinical practice, but other grafting techniques, such as inlay grafting (where the graft is the same size as the defect) and granular grafting, should also be explored to determine their effects on bone formation. As Cellnest is easy to process into various forms such as sponge, granules, films, and porous particles, generating three-dimensional transplant materials using 3D printing technology may be possible [38,54].

RGD-rich collagen contains many RGD motifs that easily bind to integrin, and thus it is characterized by its high functionality as a scaffold for cell proliferation, migration, and adhesion from the perspective of wound healing compared with other collagen materials [32,55]. Use of bone regeneration materials has evolved from simple, inert bone scaffolding materials to artificial materials containing bioactive peptides, such as peptides derived from the extracellular matrix (ECM) and bone morphogenetic protein (BMP), including bioactive peptides. Furthermore, in recent years, research has also progressed to combine structural scaffold components with biological molecules such as growth factors, amino acids, and hormones [38]. It is possible that new bone regeneration materials will be created by combining RGD-rich collagen materials such as Cellnest with bioactive substances.

## 5. Conclusions

Recombinant peptides rich in RGD motifs, derived from human type I collagen, have been shown to enhance multiple MSC functions, including proliferation, adhesion, and migration, as well as promote the expression of osteogenesis-related genes. These findings suggest their potential utility in bone regeneration for treating bone defects. Bone-forming scaffolds primarily composed of human type I collagen with RGD-rich motifs could offer a promising alternative for addressing bone defects caused by trauma, tumor resection, and congenital malformations.

## Data Availability

The raw data supporting the conclusions of this article will be made available by the authors on request.
